# Metabolic Reprogramming in Gastric Cancer: Trojan Horse Effect

**DOI:** 10.3389/fonc.2021.745209

**Published:** 2022-01-12

**Authors:** Yu-Ling Bin, Hong-Sai Hu, Feng Tian, Zhen-Hua Wen, Mei-Feng Yang, Ben-Hua Wu, Li-Sheng Wang, Jun Yao, De-Feng Li

**Affiliations:** ^1^ Department of Gastroenterology, Shenzhen People’s Hospital (The Second Clinical Medical College, Jinan University, The First Affiliated Hospital, Southern University of Science and Technology), Shenzhen, China; ^2^ Department of Rheumatology and Immunology, ZhuZhou Central Hospital, Zhuzhou, China; ^3^ Department of Gastroenterology, ZhuZhou Central Hospital, Zhuzhou, China; ^4^ Department of Hematology, Yantian District People’s Hospital, Shenzhen, China

**Keywords:** gastric cancer, glycolysis, metabolic reprogramming, tumor microenvironment, drug resistance

## Abstract

Worldwide, gastric cancer (GC) represents the fifth most common cancer for incidence and the third leading cause of death in developed countries. Despite the development of combination chemotherapies, the survival rates of GC patients remain unsatisfactory. The reprogramming of energy metabolism is a hallmark of cancer, especially increased dependence on aerobic glycolysis. In the present review, we summarized current evidence on how metabolic reprogramming in GC targets the tumor microenvironment, modulates metabolic networks and overcomes drug resistance. Preclinical and clinical studies on the combination of metabolic reprogramming targeted agents and conventional chemotherapeutics or molecularly targeted treatments [including vascular endothelial growth factor receptor (VEGFR) and HER2] and the value of biomarkers are examined. This deeper understanding of the molecular mechanisms underlying successful pharmacological combinations is crucial in finding the best-personalized treatment regimens for cancer patients.

## Introduction

Gastric cancer (GC) is currently the third leading cause of cancer-related death globally and varies significantly among different geographical areas, despite the overall morbidity and mortality are declining (1). Surgery is an effective option for the treatment of GC, while patients with advanced GC lose the best opportunity of surgery due to multiple metastasis (2). Compared with other primary tumors, GC with multiple metastases has higher tissue heterogeneity, which is caused by multiple specific gene clusters or gene mutations (3). Therefore, GC displays aggressive behavior and treatment resistance, bringing great difficulties for the development of molecular targeted drugs and individualized precise treatment. Moreover, based on the molecular classification of The Cancer Genome Atlas (TCGA), GC encompasses different molecular subtypes, such as Epstein–Barr virus (EBV 9%), microsatellite instability (MSI 22%), genomic stable (20%), and chromosomal instability (50%), and often exhibits a poor and unfavorable prognosis (4).

It has become clear enough that a single cancer hallmark (e.g., self-sufficiency in growth signals, insensitivity to antigrowth signals, evading apoptosis, limitless replicative potential, sustained angiogenesis, and tissue invasion and metastasis) cannot be used to globally define tumor alteration (5). As early as last century, Warburg found that owing to uninterrupted growth, tumor cells would reprogram their metabolism production network by circumventing mitochondrial oxidative phosphorylation and facilitating aerobic glycolysis to maintain the normal levels of ATP and NADH (6). Metabolic reprogramming, including the remodeling of glucose, lipid, glutamine, oxidative phosphorylation, and mitochondrial respiration (7), plays a pivotal role in the regulation of gene transcription, DNA damage repair, and metabolic enzymes, to transmit or release cytokines through signaling pathways in the tumor microenvironment (TME). Accumulating evidence indicates that cancer cells may transfer biologically functional molecules to their surrounding stromal cells by reprogramming metabolism, which facilitates cancer metastasis, drug resistance, and immunosuppression (8–10). If this series of cancer cells disorders are regarded as energy metabolism alteration, limiting energy currency ATP and redox currency NADH can be achieved by using small molecule drugs targeting energy metabolism or cutting off the metabolic pathway of energy supply. Similar to the Trojan horse effect, by targeting metabolic changes, we can identify potential new targets for accurate cancer treatment and design antitumor strategies to improve the concentration of drugs into cells. Therefore, metabolic reprogramming has become a promising target in cancer therapy, including refractory cancers such as GC.

Alterations in amino acid synthesis and catabolism, lipid biogenesis, and other pathways such as polyamine processing, are commonly seen in GC (11, 12). However, the development of GC and TME forms a complex loop, and the specific mechanism underlying its metabolic reprogramming remains largely unexplored. The present review outlines recent updates, addressing how bioenergetic metabolism reprogramming is involved in GC, aiming to better understand their role in the GC progression, which might help develop new therapeutic approaches by targeting GC metabolism.

## Characteristics of Metabolic Reprogramming in GC

**Figure 1:**
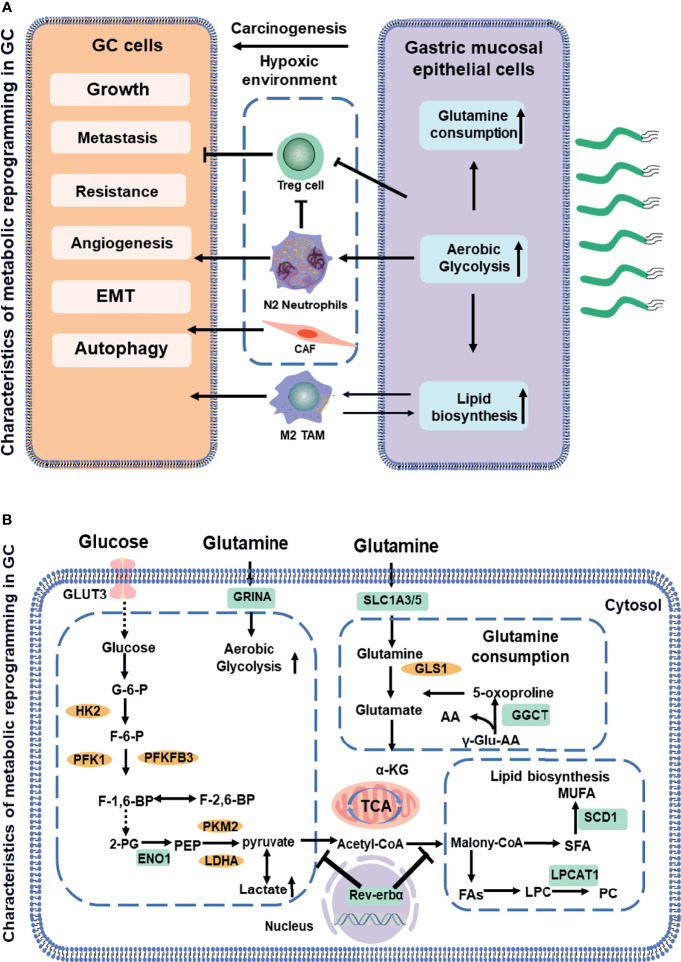
Schematic showing a comparative account of normal *vs.* cancer cell metabolic reprogramming **(A)**. The association between aerobic glycolysis (Warburg effect) and the glutamine metabolism and fatty acids metabolism. Biomarkers in GC (indicated in green boxes) along with signaling molecules (orange circles). Next, the mitochondrial dysfunction or phenotypic alteration **(B)**. AA, amino acid; CoA, coenzyme A; ENO1, enolase 1; F-6-P, fructose 6-phosphate; FA, fatty acids; G-6-P, glucose-6-phosphate; GGCT, glutamylcyclo transferase; GLUT3, glucose transporter3; GRINA, glutamate receptor; GLS, glutaminase1; HK2, hexokinase2; LDHA, lactate dehydrogenase; LPC, lysophosphatidylcholine; LPCAT1, lysophosphatidylcholine acyltransferase; MUFA, multiunsaturated fatty acid; PEP, phosphoenolpyruvate; PFK1, phosphofructokinase1; PC, phosphatidylcholine; PFKFB3, phosphofructokinase-2/fructose-2,6 bisphosphatase 3; PKM2, pyruvate kinase2; SFA, saturated fatty acids; SCD-1, stearoyl-CoA desaturase 1; TCA, tricarboxylic acid cycle. Dotted lines indicate the feed-back inhibition/regulation of some of the glycolytic enzymes by corresponding metabolites.

Malignant tumors have the common characteristics of high metabolism. However, epigenetic changes, tissue origin, differentiation status, and other internal and external factors such as oxygen and nutrients in tumor microcirculation result in a unique metabolic profile that distinguishes cancer cells from normal cells (**Table 1**). Reprogramming of the tumor metabolism includes upregulation of aerobic glycolysis, a strongly enhanced glutaminyl, and lipid accumulation in tumor cells, potentially providing energy and structural requirements for the development of cancer cells (**Figures 1A, B** **) (** 23). However, effective stratification strategies and selection of predictive biomarkers for personalized medicine are currently limited. GC, as a heterogeneous disease, lacks specific symptoms in its early stages, leading to a delayed diagnosis with three-quarters of patients presenting with non-curable advanced disease (24). Moreover, the energy metabolism reprogramming of GC has its own characteristics due to the heterogeneity. For instance, six metabolites (alanine, α-ketoisocaproic acid, proline, glycerin acid, pantothenic acid, and adenosine) show varying expression levels between GC cell lines and a normal gastric epithelial cell line (25). In particular, genome-wide expression profiles have found that an intestinal subtype of gastric tumors is involved in glucose metabolism and glutamine metabolism-related gene, and glucose transport and glucan related to metabolic genes are enriched in the diffuse subtype of GC (26). Therefore, it is urgently necessary to integrate clinical, morphological, and molecular data by identifying key metabolic processes of GC for the patient stratification for personalized therapy.

**Table 1:** Biomarker of metabolic reprogramming in GC.

	Biomarker	Function	Locations	Impactions in GC	Clinical Significance in GC
**Aerobic glycolysis**	GLUT 3 (12)	Rate-limiting glucose transport	Cytoplasm	Infiltration and polarization in GC TAM	TNM stage, DFS, OS
	ENO1 (13)	Catalyzing the conversion of 2-PG to PEP	Cytoplasm, Cell membrane	Regulation the stem cell-like characteristics	Infiltration depth, Stage, OS
	GRINA (14)	Glutamate Receptor	Membrane	Enhancing the glycolytic metabolism	Histological differentiation, TNM stage, Metastasis, Vessel invasion, perineuronal invasion
**Glutamine consumption**	SLC1A3 (15)	Glutamate transporter	Mitochondria, Nuclear	Increasing aspartate import in hypoxia	Histological differentiation, TNM stage
	GGCT (16)	Catalyzing the γ-glutamyl peptides to generates 5-oxoproline and free AAs	Cytosol, Extracellular exosome	Inhibition cell proliferation and inducing apoptosis (17)	Histological grade, LNM, TNM stage
	SLC1A5 (18)	Glutamine transporter	Plasma membrane	Inhibition of glutamine synthetase to reduce GC cell proliferation and resistance	Local invasion, LNM, TNM stages, Ki-67 expression
**Lipid biosynthesis**	SCD-1 (19)	Conversion of saturated FAs to monounsaturated FA	Endoplasmic reticulum membrane	Enhancing the tumor growth, migration, anti-ferroptosis	TNM stage, LNM, OS,
	LPCAT1 (20)	Composition of plasma membrane (21)	Endoplasmic reticulum membrane.	The conversion of LPC to PC	Tumor depth, LNM, TNM stage
	Rev-erbα (22)	Lipid metabolism nuclear receptor	Nucleus, Cytoplasm	The inhibition of proliferation by reducing glycolytic flux and PPP	TMN stage

2-PG, 2-phosphoglycerate; PEP, phosphoenolpyruvate; FAs, Fatty acids; AAs, amino acids; LNM, lymph node metastasis; PPP, pentose phosphate pathway.

### Aerobic Glycolysis

Aerobic glycolysis is the process of oxidation of glucose into pyruvate, followed by lactate production under normoxic conditions, which promotes glutaminolysis to satisfy the precursor requirements of nucleic acids (27). The upregulation of glycolysis is mostly due to the increased expressions of enzymes and transporters involved in glucose uptake, lactate production, and lactate secretion (28). **Figure 1** outlines the stepwise process of glycolysis, including the substrates and enzymes of the pathway. The glucose uptake of cells largely depends on the concentration of membrane transport proteins collectively known as the glucose transporter (GLUT) family. Significantly, GLUT 3, acting as a biomarker to determine prognosis and immune infiltration in GC, not only potentially contributes to M2 subtype transition of macrophages in the TME by mediating glucose influx (12) but also is correlated with higher tumor–node–metastasis (TNM) stage and negative survival (29). Moreover, glycolytic enzyme Enolase 1 (ENO1), as a poor prognosis biomarker in GC (13), which is involved in hypoxia, increases glucose uptake and metabolism *via* upregulating GLUT3 and promoting the lactate production (30). The molecular mechanisms of metabolic reprogramming in GC have been applied in clinical practice. For example, a study consisting of 279 patients routinely staged in the absence of metastases on CT has identified previously unsuspected metastases in 7% of patients using F-18 fluorodeoxyglucose, which would likely not have been identified by conventional staging without PET-CT in 5% (31).

### Glutamine

**Figure 2:**
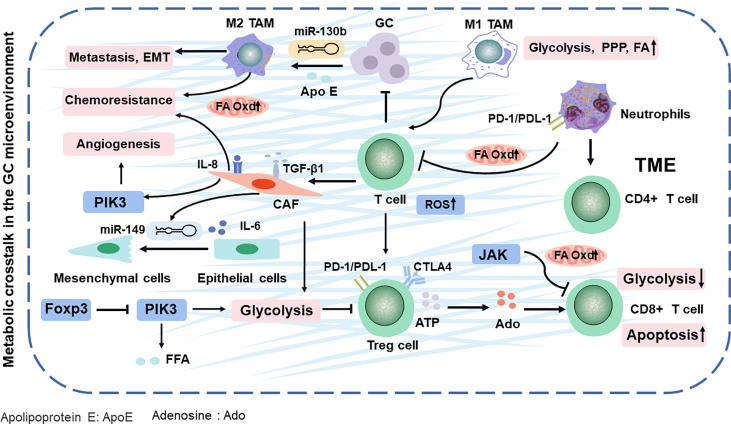
TME comprising the tumor cells and various stromal cells in GC. They evade immune surveillance during GC progression by balancing energy requirements and in TME. Finally, the metabolites of TME impacts cancer-specific or related phenotypes. Apo E, apolipoprotein E; Ado, adenosine; Oxd, oxidation; PPP, pentose phosphate pathway; ROS, reactive oxygen species; TAM, tumor-associated macrophages.

Glutamine, a new energy source for tumor cells, provides nitrogen and carbon sources that replenish tricarboxylic acid (TCA) cycle intermediates for the sake of nucleic acids. Glutamine is first converted to glutamate and ammonium by glutaminase (GLS). Subsequently, it is catalyzed by glutamate dehydrogenase (GDH) and converted to α- ketoglutarate (32). Then, α-ketoglutarate enters the TCA cycle, which provides energy and macromolecular intermediates, as seen in **Figure 2**. The combination of GLS1 and glutamyl cyclotransferase (GGCT) is highly sensitive and specific for detecting GC, which is strongly associated with histological grade, lymph node metastasis, and TNM stage (16). The SLC1 family (glutamate transporters) plays important roles in providing cells throughout the body with glutamate for metabolic purposes (33). For example, the loss of function of SLC1A3 (GLAST) and SLC1A5 (also known as ASCT2 or Na-dependent transmembrane transporter) has been implicated in the pathogenesis of GC. SLC1A3 is positively associated with the poor prognosis, and it provides a competitive advantage to GC, increasing aspartate import under the hypoxic condition (15). SLC1A5 is correlated with malignant features, such as deeper local invasion, higher lymph node metastasis, advanced TNM stages, and higher Ki-67 expression (18). However, the inhibition of glutamine synthetase remarkably reduces the proliferation and resistance of GC cells, suggesting that glutamine mediates GC growth and the therapeutic efficacy of targeted treatment (34). Interestingly, as a glutamate receptor, the N-methyl D-aspartate-associated protein 1 (GRINA) is involved in lipid and sterol synthesis (35), and it also modulates aerobic glycolysis and promotes tumor progression in GC (14).

### Fatty Acids

**Figure 3:**
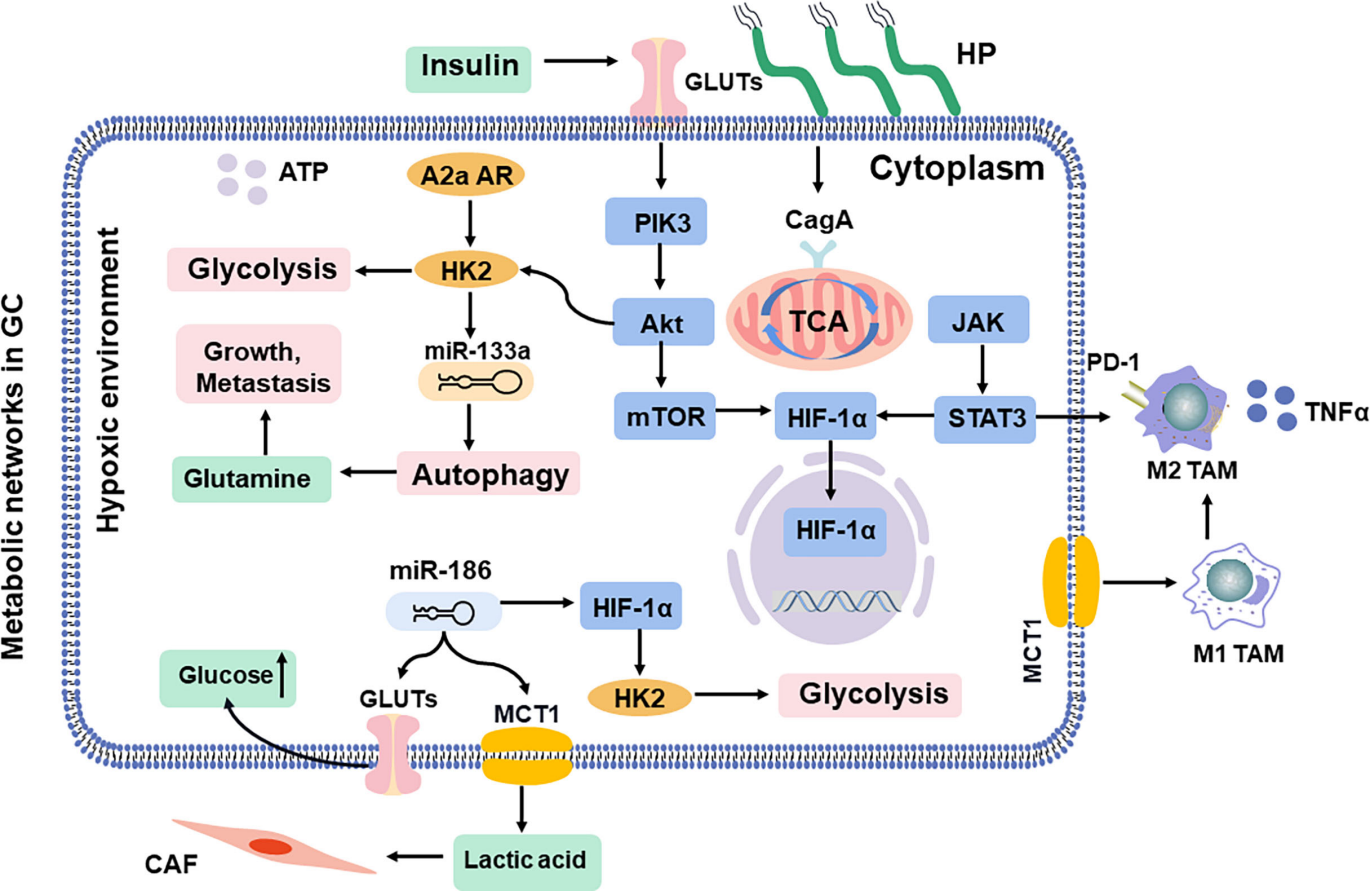
Shift in metabolic networks in GC. The metabolic intermediates of metabolic reprogramming are associated with diverse pathways in the cells inside and outside. HP, *H. pylori*; MCT, monocarboxylate channel transporter.

Fatty acids (FAs, as molecule signals and energy sources, are important as the basic backbone of many lipids and generally recognized as part of the metabolic landscape of cancer (36). The *de novo* FA synthesis pathway is enhanced to glucose and glutamine metabolism in tumor cells (**Figure 3**) **(** 11). Strikingly, FA metabolisms, FA transport, and fat differentiation-related signatures are also highly activated in GC (26). Stearoyl-CoA desaturase 1 (SCD-1), which converts saturated FAs into monounsaturated FAs, is overexpressed and exhibits the ability to promote tumor growth, migration, and anti-ferroptosis in GC (19). Lysophosphatidylcholine acyltransferase 1 (LPCAT1) is involved in the metastasis and recurrence of GC (20), especially in converting lysophosphatidylcholine (LPC) to phosphatidylcholine (PC), which is positively correlated with tumor differentiation but negatively correlated with tumor depth, lymph node metastasis, and tumor stage in GC (37). interestingly, Rev-erbα (nuclear receptor subfamily 1 group D member 1) regulates lipid metabolism nuclear receptor, and it is not only associated with TMN stages but also its reduction causes GC progression by augmenting the glycolysis (22).

Based on the TCGA dataset, a signature consisting of seven glycolysis genes (STC1, CLDN9, EFNA3, ZBTB7A, NT5E, NUP50, and CXCR4) is established, demonstrating that an immunosuppressive TME can lead to poor prognosis in GC (38). All the above evidence displays different metabolic traits compared with the tumors from which they originate, enabling survival and growth in the new TME, and it selectively and dynamically adapts their metabolism at every step during the metastatic cascade, which creates a nutrient-rich microenvironment. These alterations are pivotal to the development and maintenance of the malignant phenotype of cancer cells in unfavorable TME or metastatic sites.

## Metabolic Alteration in the GC Immune Microenvironment

TME (composed of the tumor cells, immune cells, and fibroblasts) releases various molecules or activates the metabolic reprogramming signaling in cancer cells to remodel surrounding areas (39), contributing to immune escape mechanisms and drug resistance with GC development (40). However, altered metabolism is not limited to cellular energetic pathways. For example, the metabolic programming of immune cells can affect antigen presentation, ultimately leading to the alteration of tumor immunity (**Figure 2**) **(** 41). Especially, immune-infiltrating cells in the TME can play dual roles, either promoting or inhibiting tumor growth, in response to metabolic stresses and external signals.

### T Cells

T cells have a natural ability to fight cancer cells in the TME. Yet, these cancer-fighting T cells are gradually exhausted and lose immunological memory potential (42). CD4^+^ T cells (helper T cells) and CD8^+^T cells (cytotoxic T cells) are the two broad functional groups of mature T cells (43). First, regulatory T (Treg) cells, the subsets of CD4^+^ T cells, are rapidly expanded upon encountering self-antigens expressed by cancer cells, and its accumulation in GC can decompose ATP to adenosine, then induce apoptosis, and inhibit the proliferation of CD8^+^ T cells, leading to immune inactivation and evasion (44). In addition, Treg cells can regulate transcription factor Foxp3 to restrain PIK3/Akt/mTOR signaling, which diminished glycolysis metabolism (45). Further research has demonstrated that Treg cells activate their lipid metabolism to support the survival (46). In addition, the accumulation of Treg cells in GC also activates the PI3K/Akt/mTOR pathway, which increases free fatty acids (FFAs) and generates an immunosuppressive TME, resulting in resistance to immunotherapy (47). The glycolysis and antitumor functions of CD8^+^ T cells can be inhibited by activating STAT3 to drive the FA oxidation (FAO) (48). These findings explain that the ratio of CD8^+^ T cells to Treg cells in the GC TME is an important factor for prognosis and clinical efficacies (49).

### Neutrophils

Neutrophils, as an important component of the tumor-infiltrating immune cells, can release several cytokines [such as interleukin-1β (IL-1β), tumor necrosis factor alpha (TNF-α), and interferon gamma (IFN-γ)], which is mediated by multiple mediators, including cytokines, chemokines, lipids, and growth factors in TME (50). In GC, high-infiltration neutrophils have been associated with poor prognosis (51). Especially, neutrophils in GC inhibit the proliferation of CD4^+^ T cells and form a local immunosuppressive environment through the programmed cell death 1 (PD-1)/programmed cell death protein-L1 (PDL−1) pathway (52). They secrete a wide spectrum of factors, including matrix metalloproteinases and proinflammatory cytokines, to initiate carcinogenesis (53) (**Figure 2**). Neutrophils effectively suppress normal T-cell immunity and prolong their lifespan, contributing to the migration of GC (54). In GC, neutrophils are polarized to an N2 phenotype to promote tumor migration (53). Neutrophil is often discounted as purely glycolytic (55), while oxidative neutrophils use mitochondrial FAO to produce and suppress T cells in glucose-restricted TME (56). Evidently, these results show that targeting the lipid metabolic mechanism of neutrophils and T cells can synergize with antitumor immunity.

### Tumor-Associated Macrophages

Tumor-associated macrophages (TAMs) include antitumor M1-like (M1-TAMs) or protumor M2-like (M2-TAMs) TAMs (57). Upon stimulation by IFN-γ or lipopolysaccharide (LPS), macrophages are polarized in the M1 phenotype, whereas M2 polarization can be achieved *via* incubation with IL-4 and IL-13 (58–60). The metabolic alterations of macrophage polarization can determine the phenotype and function of TAMs in promoting the cancer progression. Conversely, cancer cells can also utilize metabolic byproducts to manipulate TAMs to their benefits (61). For example, M2 macrophages are triggered by GC-derived mesenchymal stromal cells, promoting metastasis and EMT (62). Further research has found that M2 macrophage polarization from GC, involving the JAK2/STAT3 signaling pathway, is attenuated by blockading the secretion of IL-6/IL-8 (63). Most likely, M2 macrophages modulate lipid metabolism by deriving apolipoprotein E and then remodel the cytoskeleton to support migration in GC (64, 65). Especially, M2 macrophage can exacerbate the FA β-oxidation and promote the 5-fluorouracil (5-FU) chemoresistance in GC (66). The lipid restores the activity and substantially enhances the phagocytosis of TAMs, leading to promoted cytotoxic T-cell-mediated tumor regression in GC (67). In addition, miR-130b, the correspondent of the M2-TAMs in GC (68), is associated with lipid metabolism and 5-FU resistance and even can activate PI3K (69–71), which is potentially a new chemotherapeutic target by interfering immune cell metabolism in TAMs. Since TAMs have a high degree of plasticity, M2 macrophages can be repolarized to M1-TAMs. Therefore, reprogramming TAMs into antitumor activity is a new cancer treatment strategy.

### Cancer-Associated Fibroblasts

Cancer-associated fibroblasts (CAFs), a protective barrier of the tumor, activate metabolically reprogrammed TAMs (72, 73) and block T-cell penetration into tumor nests by secreting transforming growth factor beta 1 (TGF-β1) (74). It is nourished by TGF-β1, which then strongly promotes the metabolic switch from oxidative phosphorylation to aerobic glycolysis in highly metastatic GC (75, 76). Further studies show that the CAFs facilitate vasculogenic mimicry formation *via* metabolic pathways PI3K (77), which exacerbates the chemotherapeutical efficacy and prognosis of GC (78). MiR-149 links IL-6 to mediate the crosstalk between tumor cells and CAFs, leading to the enhanced epithelial-to-mesenchymal transition and stem-like properties, which alters the metabolism and allows GC cells to spread throughout the body (79, 80).

### *Helicobacter pylori* Infection

Persistent *Helicobacter pylori* infection is well-known to affect the inflammatory TME and promote GC carcinogenesis (81). In addition to involving inflammatory activation, *H. pylori* participates in various cell types, including immune cells, gastric epithelium, glands, and stem cells (82). *H. pylori* activates, polarizes, and recruits macrophages to sustain a continuous supply of proinflammatory and protumorigenic cytokines [such as IL-1, IL-6, IL-1β, TNF-α, macrophage inflammatory protein-2 (MIP-2), and inducible nitric oxide synthase (iNOS)] (83), and inevitably, they alter the metabolism as key contributors to immune evasion. The above-mentioned studies involved harnessing metabolic byproducts and hijacking the functions of tumor-infiltrating immune cells, favoring an immunosuppressive phenotype (84), which impacts many malignancy features, including the expansion and survival of tumor cells, metastasis, and angiogenesis (85). These findings provide a rationale for metabolically targeting the TME, which may assist in improving tumor responsiveness to immune checkpoint blockade (ICB) therapies. Therefore, whether the dysregulated metabolism of TME is a cell-intrinsic program or competition with GC cells for limited nutrients needs to be further discussed.

## Metabolic Networks in GC

The progression of GC involves a shared set of metabolic reprogramming pathways, which produce excess lactic acid to reduce the pH value in TME and acquire metabolic adaptations (**Figure 3**) **(** 86, 87). This metabolic alteration in GC switches from oxidative phosphorylation to glycolysis concerned promoting EMT, tumor angiogenesis, and the metastatic colonization of distant organs, resulting in regulation of the invasion-metastasis cascade (80). In addition, some pathogens, such as *H. pylori*, further mediate an inflammatory environment and trigger the oncogenic pathway, leading to DNA damage in gastric mucosal epithelial cells, continuous accumulation of intracellular abnormal metabolites, and eventually malignant transformation (88, 89).

### HIF-1α/ROS

The physiological gastrointestinal luminal epithelium is hypoxic (90), and tissue hypoxia induces metabolic reprogramming and may result in malignant transformation of gastric mucosal epithelial cells (91). Moreover, it even induces resistance to chemoradiotherapy, leading to therapeutic failure (92). Hypoxia-inducible factor-1 alpha (HIF-1α) controls the production of reactive oxygen species (ROS) in oxygen concentration, which supports the adaptation of tumor cells and mediates lactic acid efflux by the monocarboxylate channel transporter (MCT) to promote macrophage polarization in a hypoxic TME (93). In addition, insulin treatment induces glucose uptake and enhances the expression of GLUT1, which is accompanied by the apoptotic effect due to HIF-1α inhibition (94). MiR-186 is involved in the CAF formation (95), which regulates glucose uptake and lactate production *via* HIF-1α (96, 97). Approximately 70% of cases of *H. pylori* infection are involved in GC progression, which is responsible for persistent oxidative stress and DNA damage. Ultimately, HIF-1α promotes metabolic adaptation in a hypoxic environment (98). The cytotoxin-associated protein A (CagA) protein, one of the most important virulence factors of *H. pylori*, is localized in the mitochondria, where it subsequently results in a hypoxic condition in gastric epithelial cells and increases the HIF-1α activity (99). Then, the crosstalk between ROS and HIF-1α induces macrophage polarization *via* the Akt/mTOR pathway, which affects the progression of gastric lesions and state of infection (100).

### PI3K/Akt/mTOR

The PI3K/Akt/mTOR pathway is frequently activated in promoting GC aggressiveness (101). It involves enhanced aerobic glycolysis (102) and then reshapes the immunosuppressive TAMs (103). Akt, as downstream of PI3K, is an important driver of the tumor glycolytic phenotype, which stimulates ATP production to increase GLUT expression and membrane translocation, phosphorylates key glycolytic enzymes, and thereby stimulates the signal transduction of the mTOR pathway (104). Especially, the PI3K/Akt pathway is significantly activated after *H. pylori* infection in tumor cells (105). Further studies indicate that CagA protein reduces cellular amino acids, and bolstering amino acid pools prevents mTOR inhibition (106). Moreover, CagA protein activates the PI3K/Akt pathway, induces glucose metabolism, and promotes GC cell proliferation (107). It has been reported that miR-133a blocks the autophagy to ruin the abnormal glutaminolysis *via* the Akt/mTOR pathway, further inhibiting the growth and metastasis of GC (80, 108). Moreover, the A2a adenosine receptor promotes the GC Warburg effect by enhancing PI3K/Akt/mTOR pathway in hypoxic TAMs (109, 110).

### JAK/STAT

Janus kinase-signal transducer and activator of transcription (JAK/STAT) signaling, as the upstream of HIF-1α (111, 112), regulates survival and immunosuppression of GC cells and sustains inflammation in TAMs, including tumor cell recognition and tumor-driven immune escape (113–115), and it is essential in the activation of macrophages, natural killer (NK) cells, and T cells (116, 117). However, efforts to develop therapeutic STAT3 inhibitors have thus far been unsuccessful (118). Activated STAT3 upregulates energy metabolism by translocating mitochondria, which is critical for glutamate-induced cell proliferation (119). Under hypoxic conditions, STAT3 physically interacts with programmed cell death protein-L1 (PD-L1) and facilitates its nuclear translocation, enhancing the macrophage-derived TNFα-induced tumor necrosis *in vivo*, and correlates with chemotherapeutic drugs (120). Especially, *H. pylori* disrupts lipid rafts *via* JAK/STAT and thereby reduces cholesterol levels in infected gastric epithelial cells, allowing the bacteria to escape from the host inflammatory response (121). Infiltrated macrophages can release STAT3 to induce PD-L1 expression in GC, which helps tumor cells escape from cytotoxic T-cell killing and promotes the proliferation of tumor cells (122). Given that interference with STAT3 activity is an amplified signaling cascade by targeting these cytokines; it curbs the growth of GC and augments antitumor immunity (123).

Although these studies have proven many substantial crosstalks and numerous links in metabolic activities, how to allow cells to maximize growth and proliferation and activate chronically in cancer remains unknown. Beyond doubt, the precancerous lesions of gastric epithelial cells have abnormal metabolic energy, and there is a cross-relationship with the pathways mentioned above. Therefore, it seems to be more valuable to trace the heterogeneity of primary lesions and the changes in metabolic enzymes in the tumor progression. In addition, drugging a specific metabolic circuitry associated with malignancy may ultimately be efficient only on a fraction of GC cells, operating as selective pressure and favoring the rapid emergence of resistant cells.

## The Strategies of Metabolic Reprogramming in GC

Nowadays, systemic chemotherapy is still the mainstay of treatment for advanced GC. A majority of patients do not benefit from monotherapy, such as 5-FU, due to frequent relapses caused by chemotherapy-resistant cancer clones. Therefore, the 5-year overall survival rate is only 20%–35% (124–126). Accumulating evidence showed that tumor cells, in order to adapt various toxic stimuli in the TME, are involved in the mechanism of self-defense or drug resistance, including enhancing DNA damage repair capacity, increasing efflux of drugs *via* upregulated resistance-associated proteins, and upregulating antiapoptotic proteins. However, this series of activities require a large amount of ATP supply (127). Therefore, metabolic reprogramming contributes to chemoresistance. The proposed metabolic mechanisms of drug resistance involve mainly in the increase in glucose and glutamine demand, glutaminolysis and glycolysis pathways activity, promotion of reduced nicotinamide adenine dinucleotide phosphate (NADPH) from the pentose phosphate pathway, activation of FAO, and upregulation of ornithine decarboxylase for polyamine production (128). Moreover, several genes are associated with metabolic reprogramming and drug resistance, such as GLUT1, LDHA, GAPDH, MCAM, and FAO (129–132).

Currently, recurrent therapeutic resistance presents revolutionary claims, and targeting the metabolic reprogramming, such as glycolytic inhibitor, could be a strategy of Trojan Horse, which highlights the novel combinational trials and their preclinical rationale. A combination of glycolysis inhibitor and 5-FU can synergistically enhance the cytotoxicity of resistant GC cells (133). Glycolysis negatively affects survival outcomes of metastatic GC patients treated with paclitaxel-ramucirumab therapy (134).

### Molecularly Targeted Drugs

Human epidermal growth factor receptor 2 (HER2), an oncogenic tyrosine kinase, is overexpressed or amplified in 12%–20% of GC (135). Several strategies have been developed directly against HER2. However, drug resistance remains a major unresolved clinical problem (136). KU004, a HER2 inhibitor, inhibits the Warburg effect by the PI3K/Akt signaling pathway and suppresses hexokinase II (HK2), which mediates antitumor effect (137). Especially, the PI3K/Akt pathway induces targeted HER2 drug resistance in GC (138, 139). A glycolysis inhibitor MK2206 diminishes the trastuzumab resistance in HER2(+) GCs by attenuating the Warburg effect (139). Moreover, GATA6, the downstream of STAT3 (140), is involved in GC metabolic reprogramming, which may contribute to trastuzumab resistance (141). Further results indicate that Rhodium (III) complex 6, an effective STAT3 inhibitor (142), may be beneficial for targeting HER2 treatment of GC.

Aerobic glycolysis leads to the accumulation of lactate, which induces angiogenesis, an important process underlying tumor growth and metastasis (143). Ramucirumab, a vascular endothelial growth factor receptor (VEGFR) inhibitor, has shown limited benefits to GC due to metabolism activity (144). A further study suggested that glycolysis can negatively affect survival outcomes of metastatic GC patients treated with ramucirumab systemic therapy (134). Apatinib, another competitive inhibitor of VEGFR2, effectively suppresses glycolysis (145) and even induces the lipid metabolism in GC (146). The 2-deoxy-D-glucose, an inhibitor of glycolysis, can significantly reduce its angiogenic sprouting in tumor (147). PFKFB3 (glycolytic enzyme) not only regulates abnormal glycolytic metabolism in GC (148), and its inhibitors, PA-1 and PA-2, are potential antiangiogenic properties (149). Therefore, VEGFR inhibitor can be one of the cornerstones against angiogenesis therapies in GC subtypes, which represents an attractive therapeutic strategy to improve the efficacy of anti-GC treatments.

### Immunotherapy

The cancer-immunity cycle (CIC) comprises a series of events that are required for immune-mediated control of tumor growth. Interruption of one or more steps of the CIC enables tumors to evade immunosurveillance. However, attempts to restore antitumor immunity by reactivating the CIC have had limited success thus far. The suppressive activity of Treg cells is mediated by several proteins present on the cell surface, such as the cytotoxic T-lymphocyte-associated protein 4 (CTLA-4), and PD-1 (150), which induces cellular senescence and suppresses responder T cells through mediating accelerated glucose consumption (43). Immunotherapy, targeting the PD-1/PD-L1 and anticytotoxic lymphocyte antigen 4 (CTLA4) pathway, collectively named immune checkpoint inhibitor (ICI), by blocking Treg-mediated immunosuppression, derives durable remission and survival benefits for GC (151, 152). However, 50% of MSI-high GC are intrinsically resistant to PD-1 therapies (153). It is likely that continuous exposure to PD-1 antigen, which induces metabolic reprogramming of the T cell, induces T-cell exhaustion (154, 155). Diclofenac, a non-steroidal drug, turns out to inhibit the lactate transporters MCTs and improve T-cell killing, which improves the efficacy of anti-PD1 therapy (156). 6-Diazo-5-oxo-l-norleucine, a small molecule glutamine analog, increases infiltration of CD8+ T cells and sensitizes tumors to anti-PD1 therapy (157). Moreover, EBV-associated GC cells are treated with JAK2 inhibitor, PI3K inhibitor, and mTOR inhibitor, which arrests G0/G1, promotes the proliferation of T cells, and reduces the PD-L1 expression (158).

CTLA-4 represents a crucial immune checkpoint, the blockade of which can potentiate antitumor immunity. Limiting Treg cell metabolic competition in the TME may increase the effectiveness of immunotherapy (159). Especially, the effect of CTLA-4 blockade on the destabilization of T cells is dependent on T-cell glycolysis. Metformin is associated with decreased expression CTLA-4 of Treg cells, which induces glycolysis (160). Telaglenastat (CB-839), a potent GLS inhibitor, comminates with anti-PD1 or anti-CTLA4 antibodies, then increases tumor infiltration by effector T cells and improves the antitumor activity of these ICIs (161). Therefore, the combinational use of ICIs together with metabolic treatments to alleviate metabolic stress may improve the efficacy of immunotherapy.

### Natural Compounds

Natural compounds, targeting the components of mitochondria, modulate metabolic abnormalities that are a consequence of immune cell dysfunction (162, 163). For example, salazosulfapyridine blocks cystine/glutamate exchange activity and mitigates the supply of cysteine to increase intracellular ROS production, thereby increasing the effect of anticancer drugs, such as cisplatin. Especially, its combination with 2-deoxyglucose significantly inhibits cell proliferation (164). Crocin, one of the main bioactive compounds of saffron, not only inhibits the EMT, migration, and invasion of GC cells through HIF-1α signaling (165) but also protects against malignant transformation by altering mitochondrial function (166, 167). The above-mentioned results show that natural compounds have great potential in regulating metabolic reprogramming. However, there are many kinds of natural compounds and different molecular pathways, and it is still necessary to establish a huge database and screen GC cell lines with metabolic phenotype for further studies.

To sum up, several metabolic inhibitors designed to target these pathways have been advanced into preclinical trials (**Table 2**). Anticancer effect or resistance can be revered by innovative anticancer treatments targeting metabolism. Depending on tumor type, not all patients benefit from metabolic reprogramming treatment and clinical responses, and the outcome on GC progression can be either positive or negative. Therefore, understanding the mechanisms of metabolic reprogramming can be a necessary tool to identify combinations of drugs that elude resistance and allow a better response for the patients.

**Table 2:** Metabolic reprogramming drugs in GC.

	Agent	Type of metabolic reprogramming	Target pathway and protein	Observation
**Molecular targeted drugs**	MK2206	Glycolysis	PI3K/Akt	Reversion the trastuzumab resistance (139)
	Rhodium (III) complex 6	TCA cycle, glycolysis, and AA pathways	STAT3	Reversion the trastuzumab resistance (141)
	Apatinib	Glycolysis	VEGFR2/AKT1/SOX5/GLUT4	Inhibition the viability and proliferation (145)
	2-deoxy glucose	Glycolysis	JNK (168)	Inhibition the angiogenesis (147)
	PA-1, PA-2	Glycolysis	PFKFB3	Inhibition the angiogenesis (149)
**Immunotherapy**	Diclofenac	Glycolysis	MCT1, MCT4	Improvement of the anti-PD1-induced T cell killing (156)
	6-diazo-5-oxo-l-norleucine	Glycolysis	Glutamine-utilizing enzymes	Increasing infiltration of CD8+ T cells and sensitized tumors to anti-PD1 therapy (157)
	AZD1480, LY294002, rapamycin	Glycolysis	JAK2, PI3K, mTOR	Arresting the G0/G1, promoting the T-cell proliferation, reducing the PD-L1 (158)
	Metformin	Glycolysis	mTOR/AKT (169)	Decreasing expression CTLA-4 of Treg cell (160)
	Telaglenastat	Glutamine	Glutamine enzymes	Increasing effector T cells (161)
**Natural compounds**	Salazosulfapyridine	Glycolysis	Cystine/glutamate	Increasing ROS, inhibition cell proliferation (164)
	Crocin	Mitochondrial Dysfunction (170).	HIF-1α	Inhibition the EMT, migration, invasion in GC (165)

TCA, tricarboxylic acid; AA, amino acid.

## Conclusion

Historically, the numerous metabolic reprogramming advances in distinguishing tumors from adjacent, non-malignant tissues and targeting these phenotypes indicate potential clinical applications. However, most cancer metabolism research has focused on phenotypes of clinically detectable tumors or experimental models derived from them, and the metabolic reprogramming of cancer cells is much more complex than first observed. Moreover, most metabolic changes are neutral or only slightly modify cancer cell fitness under stress (171). Certain pathways are essential for the progression of selected cancers and can be exploited therapeutically, and understanding GC metabolism and identifying liabilities require a sophisticated view of how metabolic phenotypes evolve.

The development of anticancer drugs in GC presents some challenges. First is the identification of accurate biomarkers that can predict the response to anticancer therapy. The second challenge is that metabolic reprogramming has emerged as a druggable target across GC, and the clinical development of combinatorial approaches should focus on how to maximize the efficacy. Third, most of the previous metabolic reprogramming studies to this point have been focused on alterations in the metabolism of glucose, glutamine, and lipid, while metabolic reprogramming also utilizes a great variety of other microelements (126). Taken together, understanding gene alterations in metabolic reprogramming is extremely important not only for GC diagnosis and prognosis but also for the development of potential targeted therapy. We should expand the research direction from the perspective of energy metabolism reprogramming.

## Author Contributions

D-FL and Y-LB drafted the work or revised it critically for important intellectual content. H-SH, FT, ZHW, M-FY, B-HW, L-SW and JY contributed significantly to analysis and manuscript preparation. D-FL approved the final version to be published. All authors contributed to the article and approved the submitted version.

## Funding

This work was supported by the Natural Science Foundation of Guangdong Province (No. 2018A0303100024), Three Engineering Training Funds in Shenzhen (No. SYLY201718, No. SYJY201714, and No. SYLY201801), Technical Research and Development Project of Shenzhen (No. JCYJ20150403101028164, No. JCYC20170307100911479, and No. JCYJ20190807145617113), National Natural Science Foundation of China (No. 81800489), and the Natural Science Foundation of Hunan Province (No. 2021JJ70076), Technical Research and Development Project of Shenzhen (JCYJ20210324113802006).

## Conflict of Interest

The authors declare that the research was conducted in the absence of any commercial or financial relationships that could be construed as a potential conflict of interest.

## Publisher’s Note

All claims expressed in this article are solely those of the authors and do not necessarily represent those of their affiliated organizations, or those of the publisher, the editors and the reviewers. Any product that may be evaluated in this article, or claim that may be made by its manufacturer, is not guaranteed or endorsed by the publisher.
